# Athena's Pages

**Published:** 2012-04-01

**Authors:** V Raveenthiran

**Affiliations:** SRM Medical College and Hospital SRM University, Chennai, India.

Necrotizing enterocolitis (NEC) is a common surgical emergency in neonates. The list of etiological agents of NEC is already exhaustive. A recent paper published in Pediatrics adds another factor to this long list [1]. Italian researchers have shown that Ranitidine could cause NEC in neonates. Although Ranitidine has not been approved by FDA for neonates, it is frequently used off-label to manage gastro-esophageal reflux and surgical stress. In fact, Athena used to prescribe it to all neonates, including NEC patients, who are kept nil per oral with an intent to prevent mucosal ulceration. Terrin et al have shown that Athena may be wrong in her approach. They evaluated 274 neonates with very low birth weight of 401 to 1500 g. Among them 91 had received Ranitidine and 183 had not. They excluded newborn with immunodeficiency, malformations of gut, NEC prior to admission, critical illness, ranitidine therapy less than 7 days and hospitalization fewer than 8 weeks. Caregivers who determined the indication, dosage and duration of ranitidine therapy were blinded as to the nature of study. NEC was diagnosed if the clinical and radiological features correspond to Bell-Stage II or more. Other risk factors of NEC such birth weight, gestational age, Apgar score, critical risk index for babies (CRIB) score, presence of central venous access port, vascular malformation such as ductus arteriosus and mechanical ventilation have all been controlled and matched between the groups. Nearly 25% of neonates receiving Ranitidine developed sepsis as compared to only 9% who did not. Escherichia, Pseudomonos and Klebsiella infections were significantly more common in Ranitidine group. NEC occurred in 10% of newborn treated with Ranitidine while it was so in only 2% of those who were not exposed to the drug. The risk of NEC was independent of the dosage and duration of Ranitidine therapy. Mortality was 6 times higher in Ranitidine group. Athena acknowledges the logics of the study conclusion. Gastric acidity is one of the important non-immunological defense mechanisms of human body. Ranitidine induced hypochlorhydria promotes colonization of pathogenic flora in neonatal gut thereby, may predispose to sepsis and NEC. But it is perplexing as to why the two groups did not differ in the incidence of certain opportunistic pathogens such as candida, staphylococcus and group B Streptococcus. Although Athena would not call for a ban of Ranitidine in neonatal practice based on this paper, she would definitely recommend weighing the risk-benefit ratio before prescribing Ranitidine in neonates. Congenital abnormalities of kidney and urinary tract (CAKUT) are a common cause of significant mortality and morbidity in infants. CAKUT is a spectrum ranging from innocuous malformations such as supernumery kidneys to notorious posterior urethral valves. Even the most benign anomaly may be part of a syndrome spectrum and thus may determine the survival of the infant. Athena is always fascinated by prognostication and risk-stratification in CAKUT. Melo et al from Brazil have analyzed 524 newborn with CAKUT and proposed a system of risk identification for early neonatal mortality [2]. This paper is based on the registry data base of Latin-American Collaborative study of Congenital Malformation. The registry is a comprehensive data base including details of prenatal sonography, routine chromosomal analyses of all malformed babies and autopsy. Authors have excluded Exstrophy-epispadias complex from the study. During the study period of 10 years there were 29653 births. The overall prevalence of CAKUT was 18 per 1000 live births. Among them 62% were of urinary tract dilatations, 15% were renal cystic disease and 5% were renal agenesis. The remaining 18% formed the miscellaneous group comprising hypospadias, horse-shoe kidneys and so forth. Mortality was 15% in miscellaneous group while it was 54% in renal agenesis group. By univariate analysis, 6 risk factors of mortality were indentified in urinary tract dilatation group. They include first pregnancy, prematurity, renal involvement, oligohydramnios, low birth weight and associated malformation of another organ system. The last 3 factors of aforementioned list are risk factors of mortality in renal cystic disease group. Athena considers the data of this study as important source of parental counseling. Frequently obstetricians and parents ask if they can continue pregnancy when CAKUT is detected by prenatal sonography. Dilemma of a pediatric surgeon in such situation is well known. Athena has become wiser by this study that she will strongly consider medical termination of pregnancy in CAKUT especially when it is associated with renal involvement, extra-renal malformations, oligohydramnios and intrauterine growth retardation.

Primary obstructive megaureter (POM) is another malformation that has always puzzled Athena. On one hand the back pressure may progress to cause renal damage. On the other hand, the megaureter may spontaneously resolve owing to maturation of uretro-vesical junction. Athena always carved for some scientific guidance to decide whether to operate upon a primary megaureter on diagnosis or to wait for spontaneous resolution to occur. To Athena’s relief, Arena et al have published their recent finding in Scandinavian Journal of Urology and Nephrology [3]. They had followed 72 primary obstructive megaureters in 60 neonates for a mean period of 5.2 + 4.1 years (range 6 months to 15 years). Voiding cystourethrogram (VCUG) had been done in all children to rule out vesicoureteric reflux. All these neonates received prophylactic antibiotics and were monitored with periodic DTPA renogram and ultrasonography. Retrovesical cross-sectional diameter of ureter less than 6 mm was taken as end point of spontaneous resolution. Nearly 53% of them resolved over a mean period of 1.9 + 1.5 years (range 6 months to 8 years). In 25% of them ureteral dilatation persisted while in 11% ureteral dilatation significantly improved, but remained within abnormal range. Among those with persistent dilatation, 22% required ureteric reimplantation. Poor drainage in DTPA scan, Grade IV - V Hydronephrosis and ureteric diameter greater than 15 mm at diagnosis emerged as independent predictor of surgery. Athena would consider early surgical reimplantation if any one of the risk factors are present. According to the study results, most of the spontaneous resolutions have occurred within 3.6 years. Although spontaneous resolution may happen beyond this time limit, it is rare. Hence, Athena would not recommend waiting for spontaneous cure beyond 3 years of age.

Post-operative pain relief in neonates is a controversial subject. Athena, in her graduate days, was taught that neonates would not feel pain due to immaturity of nervous system. This philosophy has long been refuted. On the contrary Canadian researchers have recently shown procedural pain may permanently damage neonatal brain development [4]. They prospectively followed up 86 preterm neonates using magnetic resonance spectroscopic imaging and diffusion tensor imaging. They have also used biochemical markers such as N-acetylaspartate to choline ratio and lactate to choline ratio. They found that greater neonatal procedural pain was associated with significant reduction in white matter and subcortical grey matter of neonatal brain. Interestingly early exposure to pain affects white matter while prolonged pain exposure primarily affects subcortical grey matter. Athena will certainly be more compassionate when she operates upon a newborn in future.

Athena’s teachers might not be entirely wrong when they taught her about immature nature of neonatal nervous system. Transmission of nerve impulses depends on saltatory conduction of neuronal cell membrane. Perhaps immaturity of neonatal nerve cell membrane leads to erratic influx of sodium ions into the nerve cells. That is how Athena could explain the research findings of Leelanukrom et al [5]. These anesthesiologists from Thailand have recently shown that wound infiltration of Bupivacaine has no significant effect on post-operative pain relief in neonates and infants. Bupivacaine, like any other local anesthetics, produces anesthesia by blocking saltatory conduction of nerves. The research workers randomized 34 neonates who underwent major laparotomy into two groups; one of which received infiltration of wound with Bupivacaine while the other was control group. Neonatal Infant Pain Scale (NIPS) had been used to assess postoperative pain. If NIPS score was more than 4, fentanyl had been used for pain relief. The two groups did not differ in their NIPS scores or fentanyl requirement. Therefore, Athena would tend to rely more on systemic analgesics than local infiltration of wound. By corollary, she would question the effectiveness of caudal analgesia using local anesthetics in neonates.

Athena frequently treats jaundiced neonates. Apart from biliary atresia, she also takes care of physiological jaundice in neonates operated for a variety of conditions. Phototherapy and phenobarbitones are indispensible components of managing jaundiced neonates. Athena is recently cautioned by two studies that questioned the long-term safety of these two modalities. Kahveci et al have demonstrated the potential genotoxicity of phototherapy [6]. They estimated sister chromatid exchange in 22 neonates undergoing phototherapy. They made estimations before, during and after phototherapy as well as in late childhood (mean 3.5 years). Results of the study showed that DNA is damaged proportionate to the duration of phototherapy. However, Athena is relieved to know that this effect is only temporary. The mean sister chromatid exchange in late childhood did not differ significantly between phototherapy group and control group. In another study Bhardwaj et al have suggested that neonatal exposure to phenobarbitone may potentiate schizophrenia-like behavior in adult life [7]. It is well known that neonatal seizures increase the risk of psychiatric disorders in adulthood. Previously neurologists believed that the primary abnormality of brain that is responsible for neonatal seizures also predisposes to schizophrenia in adult life. But the authors of the paper published in Neurophramacology, by lateral thinking, hypothesize that phenobarbitone which is frequently used to treat neonatal seizures could be the culprit rather than the seizure or primary brain lesion. They tested this hypothesis in Ventral hippocampal lesion model of neonatal rats. Even in the absence of hippocampal lesion, exposure to phenobarbitone in neonatal period was found to be sufficient to disrupt sensorimotor gating. Neonatal exposure to phenobarbitone also enhanced locomotor response to amphetamine in adult rats. Athena is bewildered by the thought that some, if not many, of the biliary atresia neonates she treated with phenobarbitone may develop neuropsychiatry disorders in their adulthood. Although these two studies are preliminary, not warranting a change in practice, they certainly call for further research.

Athena is amused by an odd report of intracardiac air as a sign of necrotizing enterocolitis [8]. A 5-day-old neonate was evaluated for sudden-onset tachypnoea and desaturation. Echocardiography showed air within cardiac chambers the source of which was traced to hepatic vein. Portal vein gas is a well known criterion of Bell stage II of NEC. But this appears to be the first report describing transmigration of gas from portal vein to cardiac chamber.

## Footnotes

**Source of Support:** Nil

**Conflict of Interest:** None declared

